# The Fruit Fly *Drosophila melanogaster* as a Model System to Study Cholesterol Metabolism and Homeostasis

**DOI:** 10.1155/2011/176802

**Published:** 2011-01-20

**Authors:** Ryusuke Niwa, Yuko S. Niwa

**Affiliations:** ^1^Graduate School of Life and Environmental Sciences, University of Tsukuba, Tennoudai 1-1-1, Tsukuba, Ibaraki 305-8572, Japan; ^2^Initiative for the Promotion of Young Scientists' Independent Research, University of Tsukuba, Tennoudai 1-1-1, Tsukuba, Ibaraki 305-8577, Japan

## Abstract

Cholesterol has long been recognized for its versatile roles in influencing the biophysical properties of cell membranes and for serving as a precursor of steroid hormones. While many aspects of cholesterol biosynthesis are well understood, little is currently known about the molecular mechanisms of cholesterol metabolism and homeostasis. Recently, genetic approaches in the fruit fly, *Drosophila melanogaster*, have been successfully used for the analysis of molecular mechanisms that regulate cholesterol metabolism and homeostasis. This paper summarizes the recent studies on genes that regulate cholesterol metabolism and homeostasis, including *neverland*, *Niemann Pick type C(NPC) disease* genes, and *DHR96*.

## 1. Introduction

Cholesterol is essential for many biological processes; it plays critical roles in influencing the permeability and fluidity of cell membranes as well as in modulating the activity of intracellular signal transduction pathways through its covalent attachment to proteins such as Hedgehog [1]. Importantly, cholesterol also serves as a precursor in the synthesis of steroid hormones. Cholesterol levels are tightly controlled by the body; an overabundance of cholesterol is accompanied by a variety of prevalent diseases in humans including cardiac infarction, stroke, and neurodegenerative disorders such as Alzheimer's disease [2, 3]. Thus, the importance of cholesterol in eukaryotes and human disease pathogenesis has been intensively investigated using vertebrate model systems, and the mechanisms of cholesterol biosynthesis have been characterized in great detail. Nevertheless, our knowledge of cholesterol metabolism and homeostasis has not been fully elucidated. In particular, our knowledge of the mechanisms that regulate absorption and trafficking of dietary cholesterol is far from complete.

For many years, the fruit fly, *Drosophila melanogaster*, has served as an excellent model system for studying the mechanisms regulating essential biological processes and has had a major role in unraveling the molecular mechanisms of development and physiology [4]. The availability of genome sequences, the ease of genetic manipulation, and the large collection of available mutants make *Drosophila *an attractive system that has enabled a better understanding of human diseases at the molecular level [5]. More recently, in the past five years, studies on cholesterol homeostasis and metabolism have also been performed in this excellent genetic model organism. Similar to vertebrates, *Drosophila* requires cholesterol as a precursor for steroid hormones and as a structural component of cell membranes. In addition, the *Drosophila* genome harbors orthologs of several important regulatory genes for cholesterol homeostasis and metabolism as described below. In this paper, we will briefly summarize the studies that have used *Drosophila* to demonstrate the powerful applicability of this model in genetic studies of cholesterol biology.

## 2. Cholesterol Uptake from the Diet Is Essential for Insect Development

In vertebrates there are two major sources of cholesterol: *de novo* synthesis and dietary uptake. Especially, *de novo* cholesterol synthesis from acetate is primarily important in vertebrates [6] and tightly controlled by several proteins, including the special transcription factors called sterol-regulatory element-binding proteins (SREBPs; discussed later). In contrast, arthropods and nematodes cannot synthesize sterols from small carbon units, as their genomes lack some of genes encoding critical enzymes that are required for *de novo* synthesis [7]. Therefore, arthropods and nematodes must obtain cholesterol directly from dietary intake or from plant sterols that are then converted to cholesterol in their intestines [8]. As a corollary, the sterol content of their diets is critical for the survival of these animals. For example, the postembryonic development of *Drosophila* does not progress from the first-instar larva to the second-instar larva on a cholesterol-free diet [9] or on a low cholesterol medium [10], reflecting the needs of cholesterol for membrane lipids and steroids that are imposed by larval growth. This feature suggests that arthropods and nematodes have genetic mechanisms regulating the uptake, trafficking, and conversion of dietary cholesterol in cells. Notably, *Drosophila* on the no- or low-cholesterol diet dies in the larval stages, but not in the embryonic stage. This might be because cholesterol and/or its derivatives are maternally deposited for embryogenesis, as embryos do not uptake sterol(s) from food.

The proper regulation of cholesterol metabolism and homeostasis is critical for steroidogenesis in both vertebrates and invertebrates [11]. In arthropods, including insects, the principal steroid hormones are ecdysteroids. Among many types of ecdysteroids, ecdysone and its derivative, 20-hydroxyecdysone (20E), play indispensable roles in inducing larval molting and metamorphosis [12, 13]. Moreover, recent studies have explored the roles of ecdysone in a wider range of biological processes, such as reproduction, sleep, memory, and aging [14–16]. Ecdysteroid biosynthesis occurs in a specialized endocrine organ called the prothoracic gland (PG) during the larval stages or in ovaries of adult females. It is thought that dietary cholesterol is taken up by those steroidogenic organs and converted to ecdysone via multiple metabolizing steps [17]. In the past decade, molecular genetic studies using *Drosophila *have successfully identified several cholesterol transporters and enzymes crucial for cholesterol metabolism in the conversion step of the ecdysteroid biosynthesis pathway [18]. Below we present three examples of the evolutionarily conserved genes, whose functions in regulating cholesterol metabolism and homeostasis have been recently analyzed using *Drosophila*: *neverland*, *Niemann Pick type C (NPC) disease *genes, and *DHR96*.

## 3. *Neverland*: A Gene Encoding a Cholesterol-Metabolizing Enzyme in Ecdysteroid Biosynthesis

The great advances in the identification and characterization of ecdysteroidogenic enzymes have been accomplished through the use of a group of mutants called “Halloween mutants” [18]. These mutants exhibit morphogenetic abnormalities, such as failure of head involution and cuticle formation, and ecdysone deficiency during embryogenesis [19]. Currently, six genes responsible for Halloween mutants have been identified and characterized; five genes encode cytochrome P450 monooxygenases, and one encodes a short-chain dehydrogenase reductase [18, 20]. The temporal fluctuations of cholesterol metabolism and ecdysone biosynthesis are established in part by changes in the activities of these Halloween enzymes, which control developmental timing and homeostasis.

Besides the six Halloween enzymes, our group has identified a non-Halloween class ecdysteroidogenic gene designated as *neverland* (*nvd*). *nvd* encodes an oxygenase-like protein with a [2Fe-2S] Rieske electron carrier domain (C-X-H-X_16-17_-C-X_2_-H) that is known to function as an electron acceptor and is involved in electron transfer to other proteins [21]. We originally identified *nvd* as a gene whose expression is up-regulated in the PG during the last instar of the silkworm *Bombyx mori* [22]. *Bombyx nvd *and its *Drosophila* ortholog were predominantly expressed in PG and ovaries. Utilizing a *Drosophila* transgenic RNAi system [23], we knocked down *nvd* function only in the *Drosophila* PG cells by RNAi and showed that loss of *nvd *function caused arrest of both molting and growth. Therefore, we named the responsible gene “*neverland*” after this phenotype in which “RNAi animals cannot grow into adults.” This developmental arrest phenotype was rescued by application of 20E in food, suggesting that *nvd* plays an essential role in ecdysone biosynthesis in the PG.

More importantly, the phenotype was also rescued by application of the precursor 7-dehydrocholesterol (7dC), but not by application of cholesterol. This feeding rescue experiment strongly suggests that Nvd is required for the conversion of cholesterol to 7dC. In both insects and crustaceans, *in vitro* incubations with radio-labeled cholesterol have firmly established that the first step of ecdysteroid biosynthesis is a 7,8-dehydrogenation [17]. The Nvd proteins have strong similarities to the class IA oxygenases of prokaryotes, which possess the consensus (2Fe-2S) Rieske-type domain [24]. Therefore, it is likely that the Nvd proteins could be the cholesterol 7,8-dehydrogenases that directly catalyze the conversion of cholesterol to 7dC in ecdysteroid biosynthesis in the PG. At the same time, however, it should be noted that the 7,8-dehydrogenation has long been believed to be catalyzed by a cytochrome P450 enzyme, based on results using P450 inhibitors, such as carbon oxide and fenarimol [25]. Further studies to elucidate the enzymatic function of Nvd are currently underway.

Curiously, *nvd *orthologs are found in the genomes of animals that do not produce ecdysteroids. In the nematode *Caenorhabditis elegans*, it has been shown that the *daf-36 *gene, which encodes the ortholog of *nvd*, is required to produce the nematode steroid hormone known as dafachronic acid [26]. Dafachronic acid induces a “dauer” diapause in *C. elegans* in response to unfavorable stresses [27]. Although ecdysteroids and dafachronic acid have different steroidal structures [28], the *daf-36* mutant is rescued by feeding with 7dC but not with cholesterol, which is similar to what was observed for *nvd *in *Drosophila*. These data suggest that *nvd/daf-36 *is essential for cholesterol metabolism in both nematodes and insects. Moreover, *nvd *orthologs are conserved in other chordates, such as cidian, zebrafish, and chicken, but not in mammalian species [22]. So far, neither the conversion step of cholesterol to 7dC nor the function of *nvd *genes has been elucidated in these animals. Future studies on the *nvd *family are required for providing new insights into the conserved and divergent features among the mechanisms of cholesterol metabolism in animal species.

## 4. *NPC1 and NPC2: Drosophila* Orthologs of Human *Niemann Pick Type C (NPC) Disease* Genes

Defects in intracellular cholesterol trafficking are implicated in several human diseases, including Niemann Pick type C (NPC) disease, a fatal autosomal recessive neurodegenerative disorder. Cells from NPC disease patients show defects in intracellular lipid trafficking, and they accumulate cholesterol and other lipids within the late endosomal and lysosomal compartments [29]. The disease is caused by mutations in one of two genes, *NPC1* [30] and *NPC2 *[31], which encode a 13-transmembrane protein possessing the sterol-sensing domain and a secreted cholesterol-binding protein, respectively. In conjunction with the cholesterol accumulation phenotype of *NPC1 *or *NPC2* malfunctions in humans, as well as mice [32], it has been suggested that NPC1 and NPC2 play important roles in trafficking cholesterols through the endocytic and/or secretory pathway. However, in vertebrate models, how NPC1 and NPC2 mediate this trafficking event is far from clear.

To explore the functional role of NPC1 in cholesterol absorption and intracellular trafficking, as well as the effects of altered sterol trafficking on neuronal integrity, Drs. Leo J. Pallanck and Mathew Scott's laboratories have used *Drosophila *as a model system. There are two *NPC1* orthologs, *NPC1a* and *NPC1b*, in the *Drosophila* genome. These two laboratories independently established *Drosophila* mutants of *NPC1a* [33, 34]. They found that the most obvious phenotype of these mutants was characterized by defects in ecdysteroid biosynthesis. *NPC1a *mutants have a prolonged first instar larval stage and are unable to molt to the second instar. Consistent with this phenotype, *NPC1a* is highly expressed in the PG. Moreover, the PG-specific expression of *NPC1a *fully rescues the larval lethality of *NPC1a* mutants, suggesting a model in which NPC1a ensures the availability of sterols for ecdysteroid biosynthesis [33, 34]. The larval lethal phenotype of *NPC1a *mutants is due to a low level of ecdysteroid because *NPC1a* mutants are rescued by application of 20E in food. The mutant phenotype is also rescued by application of cholesterol or 7dC, suggesting that the ecdysteroid deficiency in the *NPC1a* mutant is caused by defects in the uptake and/or trafficking of cholesterol in the PG. As expected, *Drosophila NPC1a *mutants have aberrant accumulation of intracellular sterol. Importantly, this accumulation phenotype is similar to that seen in mammalian *NPC1 *mutants [33, 34].

The *Drosophila* genome also harbors eight orthologs of the mammalian *NPC2* family. Among them, Scott's laboratory has established mutations of two *Drosophila NPC2* orthologs, namely *NPC2a* and *NPC2b*, and investigated their phenotypes [35]. *Drosophila *double mutations of *NPC2a *and* NPC2b* are lethal during larval-pupal development and exhibit aberrant sterol accumulation defects. Like the defect in the *NPC1a* mutant, the developmental defect is also fully rescued by excess 20E, cholesterol or 7dC. Similar to what was observed for the studies of *NPC1a*, the specific expression of *NPC2 *in the PG rescues the larval lethality of *NPC2a; NPC2b *double mutants. These results suggest that NPC2 members and NPC1a are crucial for ecdysteroid biosynthesis via the regulation of cholesterol uptake and/or trafficking in the PG. Interestingly, it is suggested that mice NPC1 is also involved in steroidogenesis; *NPC1* homozygous mutant mice display neurosteroid deficiency, and the administration of supplementary allopregnanolone, a metabolite of progesterone, relieves the symptoms of NPC disease [36]. It is also noteworthy that *NPC2a; NPC2b* double mutant flies undergo apoptotic neurodegeneration, which might be analogous to the neurodegeneration in NPC disease patients [35]. Taken together, cholesterol homeostasis is a key event for steroidogenesis and neuronal development in both flies and mice. Considering that the phenotype of *NPC1a* mutants is quite similar to that of the loss of *nvd* function [22], it is possible that cholesterol uptake and/or trafficking is coupled to triggering steroid biosynthesis.

In contrast to *NPC1a*, *NPC2a,* and *NPC2b*, the other *Drosophila NPC1* family member, *NPC1b*, does not seem to be primarily involved in ecdysteroid biosynthesis in the PG. Pallanck's laboratory has shown that *Drosophila NPC1b* is expressed specifically in the midgut epithelium [37]. The tissue specificity of *NPC1b* is very different from the expression pattern of *NPC1a*, which is expressed in many types of cells [33, 34]. Pallanck's laboratory has also created *Drosophila NPC1b *mutants and has demonstrated that loss of *NPC1b* function causes a severe defect in sterol absorption, resulting in lethality at the first larval stage [37]. It should be noted that the function of *NPC1b* resembles that of an isoform of the vertebrate *NPC1* family, *NPC1L1*, which is important for intestinal cholesterol absorption in mice [38]. At the same time, surprisingly, *NPC1a*; *NPC1b *double mutants absorb sterols as efficiently as wild type animals, suggesting that there is an unknown *NPC1b*-independent mechanism in sterol absorption under *NPC1a *mutant condition [37].

In summary, the Pallanck lab and the Scott lab have uncovered the significant functional similarities of *NPC1* and *NPC2* between *Drosophila* and vertebrates. Therefore, we may be able to conclude that the *Drosophila NPC *model is useful for studying the biological functions of mammalian NPC proteins and the pathogenesis of cholesterol metabolism.

## 5. *DHR96*: A Nuclear Receptor Gene Responsible for Cholesterol Homeostasis

Transcriptional regulation is also important for cholesterol homeostasis. In vertebrates, two transcription factor families, liver X receptors (LXRs) and sterol-regulatory element-binding proteins (SREBPs), are involved in regulating cholesterol homeostasis. Vertebrate LXRs are mammalian nuclear receptors that regulate lipid metabolism upon binding to cholesterol metabolites called oxysterols [39].

The laboratories of Drs. Carl S. Thummel and Kirst King-Jones have recently reported that *DHR96*, one of the *Drosophila* nuclear receptor genes related to vertebrate *LXRs*, is essential for mediating the transcriptional response to dietary cholesterol. Like vertebrate LXRs, *Drosophila* DHR96 binds cholesterol [40]. While DHR96 is involved in regulating xenobiotic responses [41], *DHR96 *mutants do not display any apparent developmental defects when the flies are reared on normal diets. Strikingly, however, *DHR96* mutants die when grown on a low-cholesterol diet. *DHR96* mutants accumulate excess cholesterol when maintained on a high-cholesterol diet [40, 42], suggesting that *DHR96* has a role in regulating cholesterol homeostasis. Dr. King-Jones and his colleagues proposed a model in which the activity of DHR96 is elevated when cholesterol concentrations in cells drop below a critical threshold to protect cells from severe cholesterol deprivation [42]. In fact, their microarray analyses have revealed that DHR96 acts as a key regulator of the expression of the *NPC* gene family as well as of other genes that are involved in cholesterol uptake, metabolism and transport [40, 42].

The phenotypes of *DHR96* mutants are similar to those of *LXR*α** mutant mice that fail to respond properly to dietary cholesterol and accumulate hepatic cholesterol when maintained on a high-cholesterol diet [43]. LXRs also control a number of genes related to DHR96-regulated genes, including* NPC1* and *NPC2*, in response to dietary cholesterol in mice [44]. These results strongly indicate that vertebrate LXRs and *Drosophila* DHR96 play similar regulatory roles in controlling cholesterol homeostasis. 

As mentioned above, many studies have identified the SREBP family of transcription factors as critical regulators of fatty acid and cholesterol biosynthesis pathways [45]. Vertebrates SREBPs are activated by proteolysis in response to declines in cellular cholesterol levels. Subsequently, the cleaved SREBPs are imported to the nucleus and act as transcription factors. All genes known to be necessary for cholesterol biosynthesis have been suggested as putative SREBP target genes [46]. In the *Drosophila* genome, a single definite ortholog of the *SREBP* gene has been found. However, *Drosophila* SREBP does not seem to be involved in cholesterol homeostasis because its activity is regulated by palmitate, but not by sterols [47].

## 6. Future Perspectives

The findings described above emphasize the similarity of the pathways that regulate cholesterol metabolism and homeostasis among animal phyla. At least four families of genes, namely *neverland*, *NPC1*, *NPC2,* and *DHR96*, are shared by vertebrates and *Drosophila*. Moreover, the *Drosophila* genome encodes other genes that are orthologs of vertebrate regulators of cholesterol including low-density lipoprotein receptors, ABC transporters, acyl coenzyme A/cholesterol acyltransferase genes, and a steroidogenic acute regulatory (StAR) protein. It has been demonstrated that the *Drosophila Start1* gene, an ortholog of the vertebrate *StAR protein* gene, is predominantly expressed in the PG [48]. Because vertebrate StAR proteins are involved in cholesterol traffic and steroid synthesis [49], *Drosophila Start1* could have an important role in ecdysteroid biosynthesis via cholesterol trafficking. Another study using *Drosophila *has also revealed that the trafficking of 7dC in the PG is regulated by a gene called *ecdysoneless* that encodes a protein that is well conserved throughout eukaryotes [50, 51]. The expression of some of these genes is affected by DHR96 and/or exposure to a high-cholesterol diet [42], implying that these genes may have important roles in cholesterol metabolism and homeostasis. To further analyze the functions of these conserved genes, a genetic approach in *Drosophila* could prove to be a powerful alternative to biochemical and cell biological approaches in vertebrates.
